# A type 2 cytokine axis for thymus emigration

**DOI:** 10.1084/jem.20170271

**Published:** 2017-08-07

**Authors:** Andrea J. White, Song Baik, Sonia M. Parnell, Amanda M. Holland, Frank Brombacher, William E. Jenkinson, Graham Anderson

**Affiliations:** 1 Institute for Immunology and Immunotherapy, College of Medical and Dental Sciences, Medical School, University of Birmingham, Birmingham, England, UK; 2 International Centre for Genetic Engineering and Biotechnology, Cape Town Component, Institute of Infectious Diseases and Molecular Medicine and South African Medical Research Council, University of Cape Town, Cape Town, South Africa

## Abstract

In the thymus, stromal microenvironments support a developmental program that generates mature T cells ready for thymic exit. The cellular and molecular specialization within thymic stromal cells that enables their regulation of specific stages of thymocyte development is poorly understood. Here, we show the thymic microenvironment expresses the type 2 IL-4R complex and is functionally responsive to its known ligands, IL-4 and IL-13. Absence of IL-4Rα limits thymocyte emigration, leading to an intrathymic accumulation of mature thymocytes within medullary perivascular spaces and reduced numbers of recent thymic emigrants. Thymus transplantation shows this requirement maps to IL-4Rα expression by stromal cells, and we provide evidence that it regulates thymic exit via a process distinct from S1P-mediated migration. Finally, we reveal a cellular mechanism by which IL-4^+^IL-13^+^ invariant NKT cells are necessary for IL-4Rα signaling that regulates thymic exit. Collectively, we define a new axis for thymic emigration involving stimulation of the thymic microenvironment via type 2 cytokines from innate T cells.

## Abstract (precis)

White et al. describe a new mechanism of thymus emigration that is controlled by expression of the type 2 IL-4 receptor by thymic stroma and production of IL-4 and IL-13 by thymic-resident invariant NKT cells.

## Introduction

Thymic organization and the availability of distinct cortical and medullary intrathymic microenvironments provide a specialized framework that guides developing thymocytes through multiple stages of migration, proliferation, and differentiation (Takahama, 2006; Boehm, 2008). Importantly, understanding mechanisms that control intrathymic T cell development requires identification of stromal cell–expressed regulators that mediate specific developmental events. For example, restricted expression of DLL4, CD83, β5t, and CXCL12 to the cortex (Plotkin et al., 2003; Murata et al., 2007; Hozumi et al., 2008; Koch et al., 2008; Liu et al., 2016; von Rohrscheidt et al., 2016) enables this site to mediate CD4^−^CD8^−^ (double-negative [DN]) T cell commitment, preTCR–mediated maturation and positive selection of CD4^+^CD8^+^ double positive (DP) thymocytes. Similarly, expression of Aire, costimulatory molecules and CCL19 and CCL21 (Degermann et al., 1994; Anderson et al., 2002; Ueno et al., 2004) in the medulla creates a site for tolerance induction and postselection development and migration (Cowan et al., 2013; Webb et al., 2016; Xing et al., 2016). Thus, correct positioning of immature DP thymocytes in the cortex and mature single-positive (SP) thymocytes in the medulla regulates intrathymic T cell development.

Few known factors control functional specialization of thymic microenvironments. Consequently, differing roles of stromal cells in thymocyte development are poorly understood, and thus, the identification of novel regulators of thymic stroma is essential in understanding thymic control of T cell production. Here, we show that the cytokine receptor component IL-4Rα is expressed in the thymus medulla, including a subset of medullary thymic epithelial cell (TEC [mTEC]), where it forms part of a functionally active type-2 IL-4R complex. Analysis of T cell development in *Il4ra^−/−^* mice revealed defects in thymus emigration that map to expression of IL-4Rα by the thymic microenvironment. We provide evidence that IL-4Rα influences thymic egress via a mechanism distinct from the S1P–S1P_1_ axis and identify CD1d-restricted invariant NKT (iNKT) cells as key regulators of emigration by providing IL-4 and IL-13 to trigger type-2 IL-4R signaling. Collectively, type-2 cytokines from innate T cells are a novel component of mechanisms controlling αβT cell egress from the thymus.

## Results and discussion

### Thymus medullary disorganization in *Il4ra^−/−^* mice

**Figure 1.**
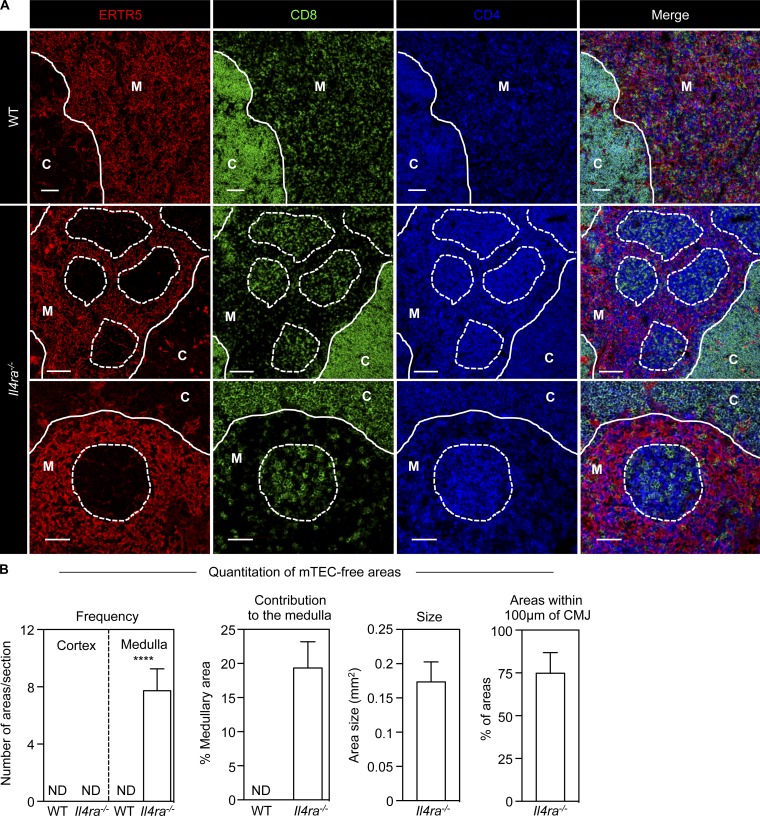
**Mature thymocytes accumulate in the thymic medulla of *Il4ra^−/−^* mice.** (A) Images of 10-wk-old WT and *Il4ra^−/−^* mice thymus. C, cortex; M, medulla; continuous line is the CMJ; dotted lines, boundaries of mTEC-deficient areas of SP thymocyte accumulation. Scale bars in WT and top row of *Il4ra^−/−^* images, 100 µm; bars in the bottom row of *Il4ra^−/−^* images, 50 µm. Images representative of *n* = 10 mice from three experiments. (B) Quantitation of mTEC-free areas, three to four randomly chosen sections from each mouse were analyzed, *n* = 4 in two separate experiments. ND, not detected. Error bars indicate SEM; a Mann-Whitney nonparametric *U* test was performed; ****, P < 0.0001.

To identify new regulators of thymus function, we analyzed tissue organization and thymocyte distribution in thymic sections from mutant mice in which thymocyte–stromal cross talk may be disrupted. Mice lacking IL-4Rα (*Il4ra^−/−^* mice) had disorganization of the thymic medulla, which contained epithelial-free areas lacking ERTR5^+^ mTEC (Fig. 1). Interestingly, these areas were not acellular cysts but contained mature SP4 and SP8 thymocytes (Fig. 1 A), including SP4 Foxp3^+^ Tregs (not depicted). Quantitative analysis showed individual *Il4ra*^−/−^ thymic sections contained ∼8 mTEC-free areas with a mean size of 0.15 mm^2^, contributing to 20% of the total medulla area (Fig. 1 B). Approximately 75% of these areas were located within 100 µm of the corticomedullary junction (CMJ; Fig. 1 B), a site of thymic egress (Weinreich and Hogquist, 2008; Maeda et al., 2014). Despite these abnormalities, cortex–medulla separation remained intact, as did typical localization of DP and SP thymocytes in the cortex and medulla areas (Fig. 1 A). Although medulla disorganization can be caused by altered mTEC development (Boehm et al., 2003), cortical TEC (cTEC) and mTEC^lo^/mTEC^hi^ subsets were comparable in WT and *Il4ra^−/−^* mice (Fig. S1). Thus, absence of IL-4Rα causes alterations in the medullary distribution of SP thymocytes that are not explained by altered TEC development.

### IL-4Rα regulates thymocyte egress

**Figure 2.**
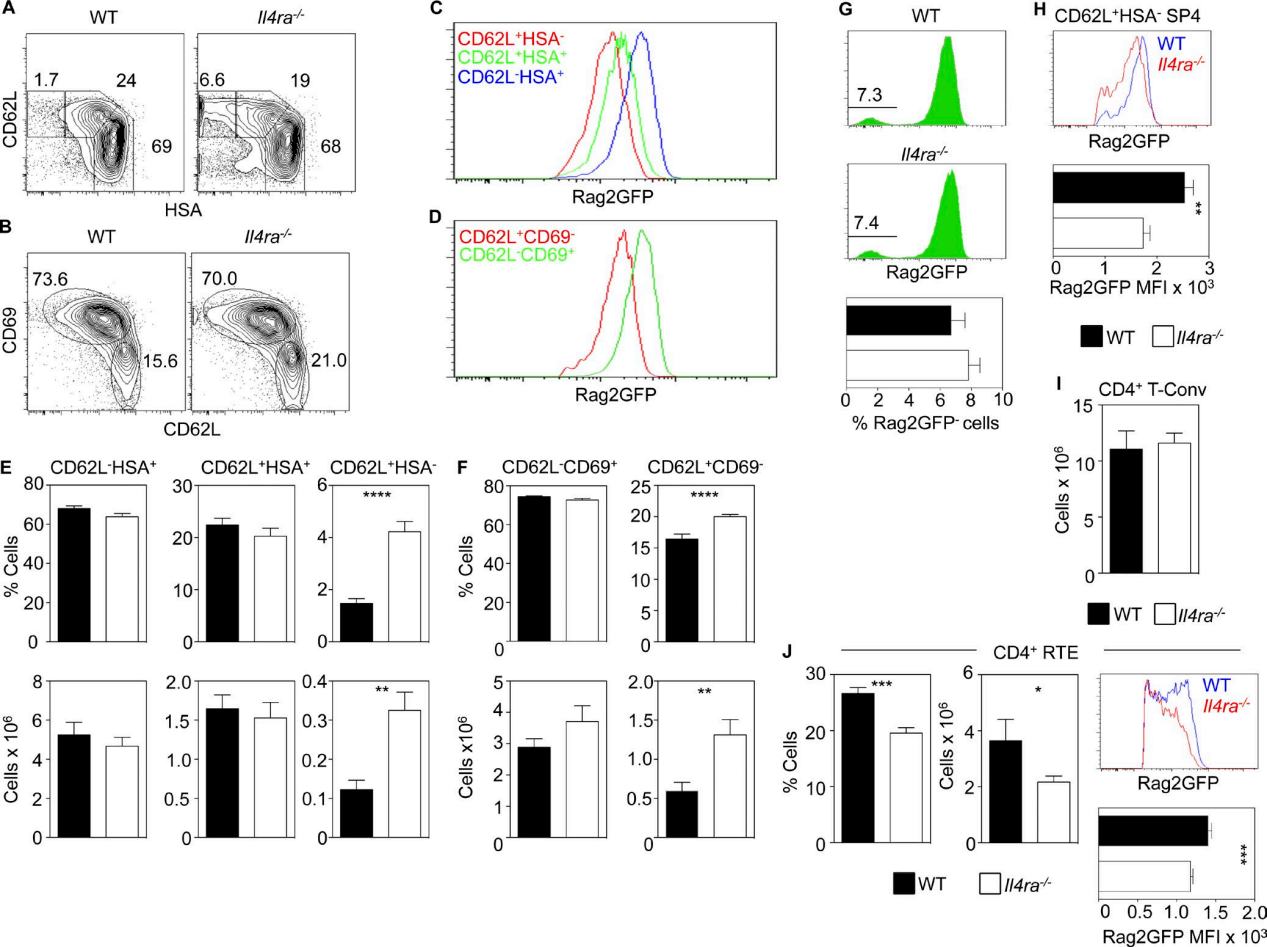
**IL-4Rα regulates thymic egress.** (A and B) TCRβ^+^PBS57^−^CD44^−^Foxp3^−^ SP4 T-conv thymocytes from WT and *Il4ra^−/−^* mice. (C and D) Rag2GFP levels in indicated SP4 thymocyte subsets from WT mice. (E and F) SP4 T-conv quantitation in WT and *Il4ra^−/−^* mice. (G) Rag2GFP in SP4 T-conv from WT and *Il4ra^−/−^* mice and frequency of GFP^−^ cells in WT and *Il4ra^−/−^* mice. (H) Rag2GFP in conventional SP4 thymocytes from WT (blue) and *Il4ra^−/−^* (red) mice with mean fluorescence intensity (MFI) of Rag2GFP. (I) Splenic CD4^+^Foxp3^−^ T cells in WT and *Il4ra^−/−^* mice. (J) Frequency, number, and Rag2GFP levels in splenic WT and *Il4ra^−/−^* GFP^+^ RTE. All data represent at least three experiments, *n* = 10 for both WT and *Il4ra^−/−^* mice. Error bars indicate SEM. A Mann-Whitney nonparametric *U* test was performed; *, P < 0.05; **, P < 0.01; ***, P < 0.001; ****, P < 0.0001.

Further analysis of the thymic defect in *Il4ra^−/−^* mice showed all major thymocyte subsets (DN and DP precursors and mature SP4 thymocytes and Foxp3^+^ Tregs) were present. We also saw the reported reduction in SP8 thymocytes caused by loss of IL-4-dependent eomesodermin^+^ innate SP8 cells (not depicted; Jameson et al., 2015). To perform detailed thymocyte analysis, we focused on conventional SP4 thymocytes (SP4 T-conv), defined here as CD44^−^mCD1dPBS57^−^Foxp3^−^ to exclude recirculating T cells, iNKT cells, and Tregs. When we separated SP4 T-conv thymocytes using CD62L and heat-stable antigen (HSA), we saw three distinct subsets (Fig. 2 A) that were Rag2GFP^+^ but which had progressively lowering levels of Rag2GFP (Fig. 2 C). Thus, and consistent with a previous study (Mouri et al., 2014), CD62L/HSA can be used to identify sequential stages (least-mature CD62L^−^HSA^+^, then CD62L^+^HSA^+^, then most-mature CD62L^+^HSA^−^) in SP4 T-conv maturation (Mouri et al., 2014). Although immature CD62L^−^HSA^+^ and CD62L^+^HSA^+^ SP4 T-convs were unaltered, both the frequency and number of mature CD62L^+^HSA^−^ SP4 T-convs were increased in *Il4ra^−/−^* mice (Fig. 2, A and E). In a complementary approach (Boehm et al., 2003), we used CD62L/CD69 to examine developmental stages in SP4 T-convs. Again, and consistent with the usefulness of this approach to identify sequential stages in SP4 thymocyte development, CD62L^−^CD69^+^ and CD62L^+^CD69^−^ subsets were Rag2GFP^+^ and showed progressively decreasing levels of Rag2GFP (Fig. 2, B and D), indicating transition through immature CD62L^−^CD69^+^ and then mature CD62L^+^CD69^−^ stages. Importantly, using this second approach, *Il4ra*^−/−^ mice had increased frequencies and numbers of the most mature CD62L^+^CD69^−^ SP4 cells (Fig. 2, B and F). Thus, two complementary approaches demonstrate an increase in the most-mature SP4 T-conv thymocytes in *Il4ra^−/−^* mice.

As increased SP thymocytes can be caused by altered thymic egress (Boehm et al., 2003), we looked for perturbation of this process. In Rag2GFP mice, GFP levels indicate thymocyte medullary dwell time and discriminate developing thymocytes from recirculating mature GFP^−^ T cells. Moreover, unlike intrathymic FITC injection, GFP directly identifies recent thymic emigrants in a noninvasive manner and so avoids possible confounding side effects caused by surgical intervention (Boursalian et al., 2004; McCaughtry et al., 2007; Hauri-Hohl et al., 2014; Cowan et al., 2016). In WT Rag2GFP and *Il4ra^−/−^* Rag2GFP mice, the frequency of GFP^−^ SP4 thymic cells was comparable (Fig. 2 G), indicating increased mature cells in the *Il4ra^−/−^* thymus is not due to enhanced peripheral T cell recirculation. Interestingly, GFP levels in mature CD62L^+^HSA^−^ SP4 T-convs were significantly lower in *Il4ra^−/−^* mice compared with WT mice (Fig. 2 H). Moreover, although numbers of splenic SP4 T cells in *Il4ra^−/−^* mice were unaltered (Fig. 2 I), we saw a significant reduction in GFP^+^ SP4 recent thymic emigrants (RTEs). These cells also had significantly lower GFP levels compared with WT, indicating a more-mature status (Fig. 2 J). Collectively, these data suggest that, in *Il4ra^−/−^* mice, prolonged medullary occupancy of mature SP4 T-conv leads to their intrathymic accumulation, which is reflected by reduced efficacy of thymic egress and diminished RTE numbers.

**Figure 3.**
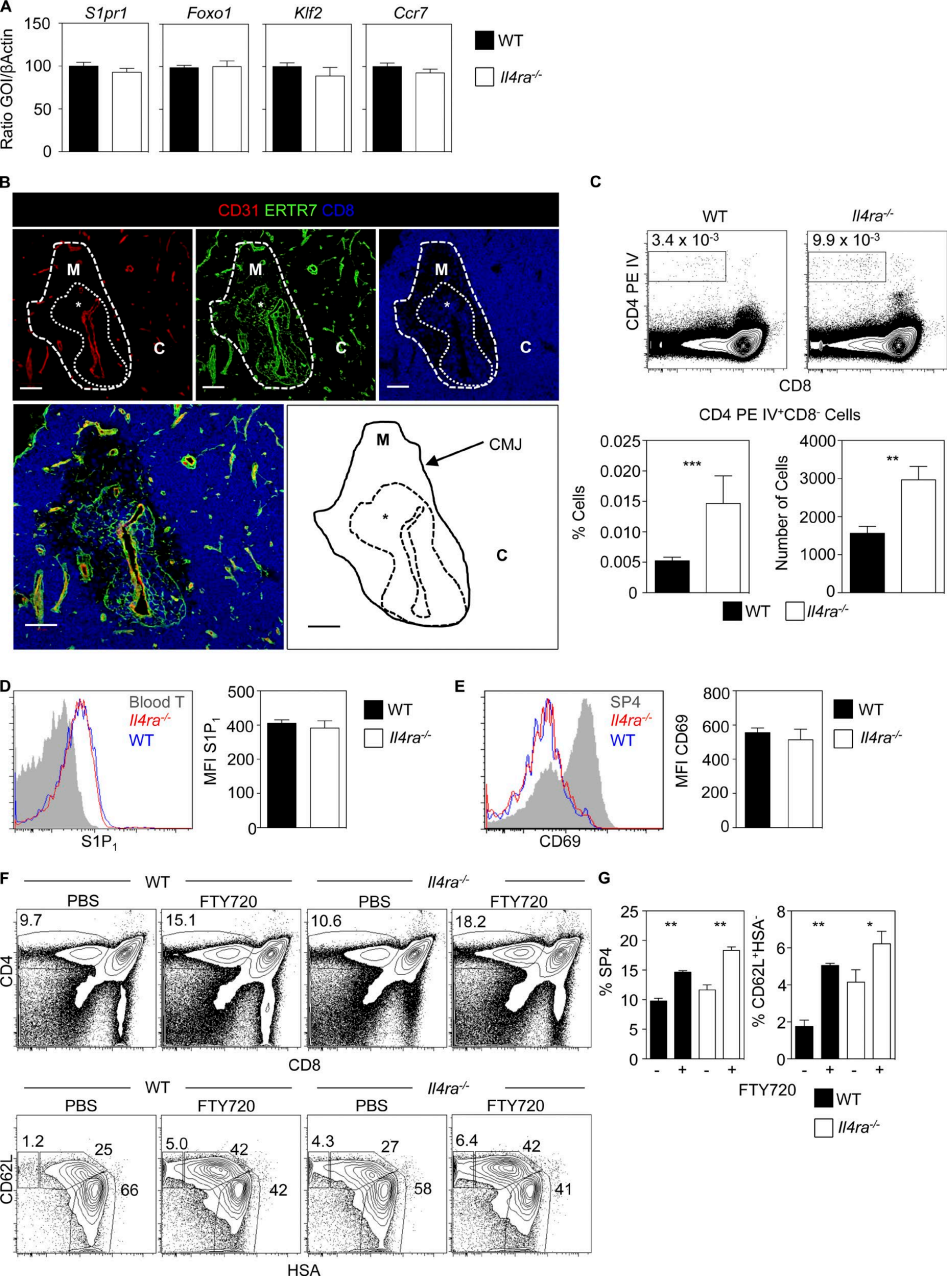
**The S1P–S1P_1_ axis remains active in *Il4ra^−/−^* mice.** (A) Quantitative PCR of CD62L^+^HSA^−^ SP4 T-conv from WT and *Il4ra^−/−^* mice. mRNA levels were normalized to β-actin; error bars indicate SEM, and data are typical of three independent experiments. (B) Thymic sections from *Il4ra*^−/−^ mice stained for CD31, ERTR7, and CD8. C, cortex; M, medulla; *, PVS. Data represent three experiments, *n* = 4. Bars, 100 μm. (C) Flow cytometry of thymocytes from WT and *Il4ra^−/−^* mice i.v. injected with anti-CD4PE (top). Quantitation of anti-CD4PE–labeled SP4 cells in WT and *Il4ra*^−/−^ mice (bottom); *n* = 6 from two separate experiments. Surface levels of S1P_1_ (D) and CD69 (E) in conventional SP4 thymocytes from WT and *Il4ra^−/−^* mice. (D) Gray histogram is peripheral blood CD4^+^ T cells; data represent at least three experiments, *n* = 6. (E) Gray histogram is CD69 on total SP4 thymocytes, Data represent at least three experiments, *n* = 10 for WT and *Il4ra^−/−^* mice. (F) Effects of FTY720 in WT and *Il4ra^−/−^* mice. (Top) CD4/CD8 in total thymocytes. (Bottom) CD62L/HSA in conventional SP4 thymocytes. (G) Proportions of total SP4 and CD62L^+^HSA^+^ SP4 T-conv. Data represent at least two experiments, *n* = 8. Error bars indicate SEM. A Mann-Whitney nonparametric *U* test was performed. *, P < 0.05; **, P < 0.01; ***, P < 0.001.

SP thymocytes undergo maturation events in the medulla, including KLF2-dependent up-regulation of S1P_1_, a cell surface receptor for sphingosine-1-phosphate (S1P) that influences thymic exit (Allende et al., 2004; Matloubian et al., 2004; Carlson et al., 2006; Weinreich and Hogquist, 2008). We found that mature SP4 T-convs from *Il4ra^−/−^* mice had an intact “thymic exit” phenotype, expressing normal levels of mRNAs for S1P_1_ and KLF2 (Fig. 3 A). Levels of CCR7 and Foxo1, representing additional regulators of thymic egress (Ueno et al., 2002; Gubbels Bupp et al., 2009), were also unaffected (Fig. 3 A). Thus, postselection maturation of *Il4ra^−/−^* SP T-convs occurs normally, indicating their intrathymic accumulation is not caused by an inability to acquire the emigration machinery for exit.

As thymic exit involves transit through the perivascular space (PVS) that surrounds blood vessels (Mori et al., 2007; Zachariah and Cyster, 2010), we further examined SP thymocyte accumulations in tissue sections from *Il4ra^−/−^* mice. Thymocyte accumulations were detected around CD31^+^ blood vessels, in between ERTR7^+^ basement membrane layers, suggesting that medullary abnormalities in *Il4ra^−/−^* mice are caused by increased thymocyte accumulations within enlarged PVS (Fig. 3 B). To directly assess that, we i.v. injected anti-CD4 antibodies to label cells in the thymic PVS (Zachariah and Cyster, 2010; Mouri et al., 2014). *Il4ra^−/−^* mice contained significantly higher proportions and numbers of SP4 thymocytes labeled with the injected antibody (Fig. 3 C). Thus, the increase in SP thymocytes in *Il4ra*^−/−^ mice is accompanied by their increased accumulation within thymic PVS, indicating a role for IL-4Rα in thymic egress.

Given the importance of S1P/S1P_1_ in thymic emigration, we looked for evidence for perturbation of this axis in *Il4ra^−/−^* mice. Thymic stroma and DCs (Bréart et al., 2011; Cyster and Schwab, 2012; Zamora-Pineda et al., 2016) keep thymic S1P levels low, and increased S1P levels reduce thymocyte S1P_1_ expression, which inhibits egress (Lo et al., 2005; Schwab et al., 2005; Pappu et al., 2007; Bréart et al., 2011). Consequently, alterations in cell surface S1P_1_ indicate altered intrathymic S1P concentrations. Similarly, altered S1P levels can be revealed by measuring expression of CD69 (Bréart et al., 2011), a molecule that associates with, and is down-regulated by, S1P_1_. Importantly, levels of both S1P_1_ and CD69 were comparable in WT and *Il4ra^−/−^* SP4 T-convs (Fig. 3, D and E). Because S1P-mediated thymocyte emigration is inhibited in vivo by FTY720, an immunosuppressant that binds to and causes S1P_1_ internalization (Matloubian et al., 2004; Maeda et al., 2014), we compared its effects in WT and *Il4ra^−/−^* mice. As expected, FTY720 increased SP4 thymocytes in WT mice, notably mature CD62L^+^HSA^−^ cells (Fig. 3, F and G). Importantly, *Il4ra^−/−^* mice responded similarly to FTY720, with a significant relative increase in mature CD62L^+^HSA^−^ SP4 T-convs (Fig. 3, F and G). Thus, lack of perturbation of S1P_1_ and CD69 in SP4 thymocytes from *Il4ra^−/−^* mice, together with receptiveness to FTY720-mediated blockade of thymic egress, indicates their thymic alterations are not due to an underlying dysregulation of S1P-mediated migration. Rather, these findings point toward a distinct, additive mechanism, in which IL-4Rα regulates thymic egress.

### Expression of type 2 IL-4R by medullary thymic epithelium

**Figure 4.**
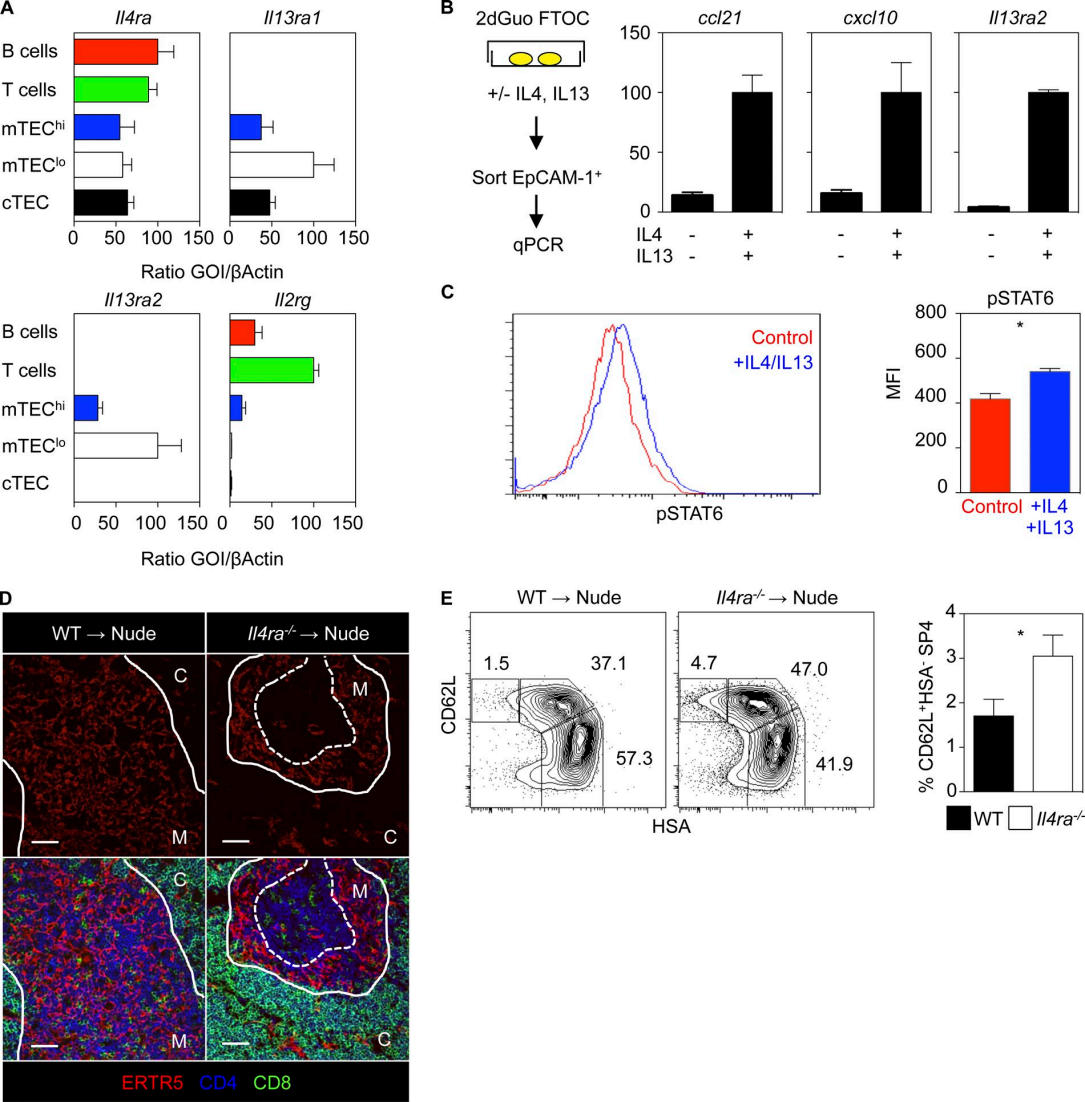
**mTECs express the type 2 IL-4R.** (A) Quantitative PCR of cells from adult WT mouse thymus and spleen, error bars indicate SEM, and data are typical of three independent experiments. (B) Quantitative PCR of sorted EpCAM1^+^ TECs from WT 2dGuo FTOC ± IL-4/IL-13 for 4 d. mRNA levels were normalized to β-actin; error bars indicate SEM, and data are typical of three independent experiments. (C) Untreated and IL-4/IL-13–stimulated EpCAM1^+^ TEC from 2dGuo FTOC stained with anti-phospho-STAT6, *n* = 4 from three independent experiments. (D) Confocal images of WT and *Il4ra^−/−^* mice 2dGuo FTOC 10-wk-old thymus transplants; data represent at least two experiments, *n* = 6 for WT and *Il4ra^−/−^* mice. Bars, 50 μm. (E) Analysis of conventional SP4 thymocytes from WT and *Il4ra^−/−^* thymic grafts; data represent at least two experiments, and *n* = 6 for WT and *Il4ra^−/−^* mice. Error bars indicate SEM. A Mann-Whitney nonparametric *U* test was performed. *, P < 0.05.

Although pairing of IL-4Rα with the common γ-chain (γc) on hematopoietic cells forms the type-1 IL-4R that binds IL-4, pairing with IL-13Rα1 in epithelial cells forms the type-2 IL-4R that binds both IL-4 and IL-13 (Kawashima et al., 2006; LaPorte et al., 2008; Gour and Wills-Karp, 2015). Consistent with the idea that the type 2 IL-4R may influence thymus function, we saw mTEC-free areas containing SP thymocytes and altered thymic egress in both *Il4^−/−^* and *Il13^−/−^* mice (Fig. S2, A and B). Interestingly, we found that TEC expressed both *Il4ra* and *Il13ra1,* with the highest levels in mTEC^lo^ (Fig. 4 A). mTEC^lo^ also expressed the highest levels of *Il13ra2* (Fig. 4 A), encoding a soluble decoy receptor for IL-13, which is induced by type 2 IL-4R signaling (Jakubzick et al., 2003). To see whether this pattern of gene expression correlated with functional expression of the type-2 IL-4R by TEC, we cultured alymphoid 2-deoxyguanosine (2dGuo) WT fetal thymus organ culture (FTOC) with IL-4 and IL-13, then isolated EpCAM1^+^ TEC. Strikingly, IL-4/IL-13 induced *Il13ra2* mRNA expression in TEC (Fig. 4 B). Moreover, TEC isolated from IL-4/IL-13–treated 2dGuo FTOC also showed strong induction of the chemokines *Ccl21* and *Cxcl10* (Fig. 4 B), both known products of mTEC (Drennan et al., 2009; Lkhagvasuren et al., 2013). Finally, flow cytometric analysis of EpCAM1^+^ TEC using an anti-phospho-STAT6 antibody showed that, compared with untreated cells, TEC treated with IL-4 and IL-13 had a significantly increased level of phospho-STAT6 expression (Fig. 4 C). Thus, TEC represents at least one thymic stromal cell type that is responsive to type-2 IL-4R ligands.

To see whether defects in thymus emigration in *Il4ra^−/−^* mice map to a requirement for IL-4Rα expression by the thymic microenvironment, we transplanted 2dGuo-treated FTOC from *Il4ra^−/−^* mice into nude mice. Here, IL-4Rα expression by host-derived hematopoietic and nonthymic stromal cells remains intact. Importantly, analysis of grafts showed that *Il4ra^−/−^* thymuses contained intrathymic accumulations of SP thymocytes within mTEC-free areas (Fig. 4 D) and had increased mature CD62L^+^HSA^−^ SP4 cells (Fig. 4 E). Together, expression of *Il4ra*, *Il13ra1*, and *Il13ra2* by TEC, and their responsiveness to exogenous IL-4/IL-13 stimulation in vitro, provides evidence for their expression of a functional type-2 IL-4R complex. In addition, data from *Il4ra^−/−^* thymus transplant experiments indicates that normal thymic emigration requires type-2 IL-4R expression by the thymic microenvironment.

### iNKT cells produce type 2 cytokines for thymus emigration

**Figure 5.**
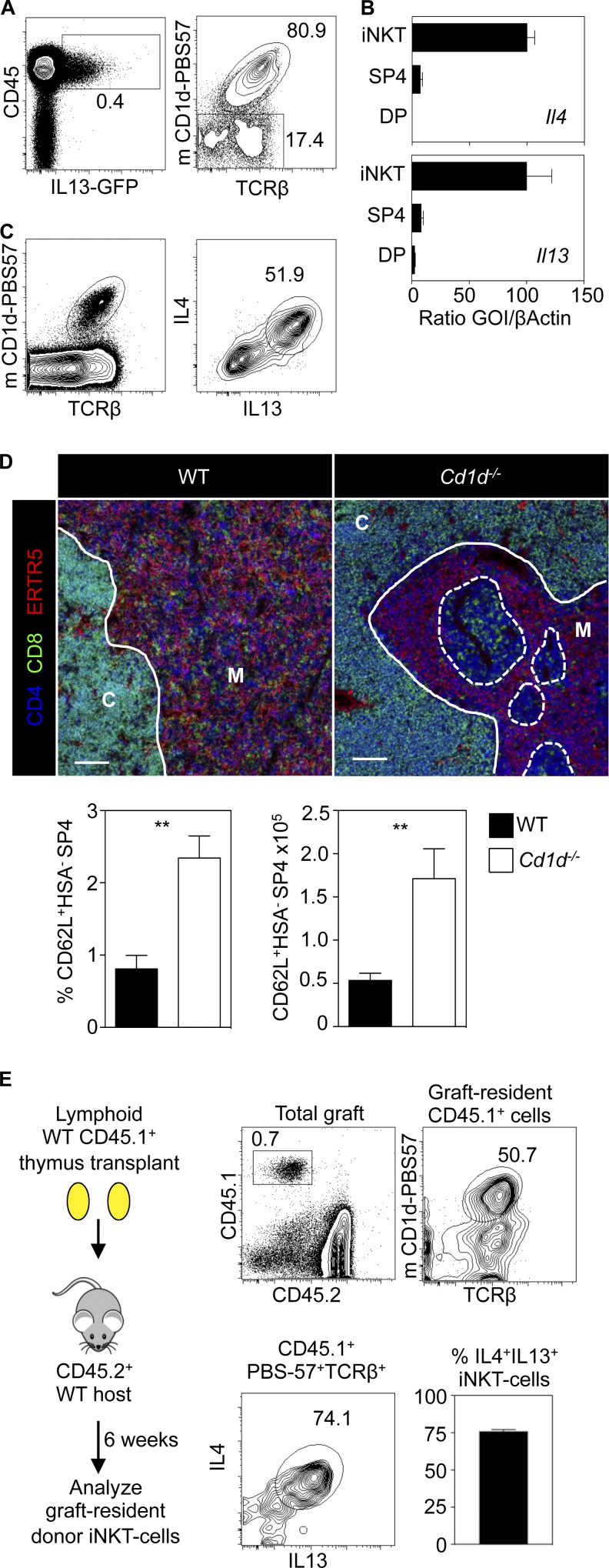
**Type 2 cytokines from iNKT cells regulate thymic egress.** (A) CD45/IL-13GFP in thymus cells from IL-13GFP mice. (Right) PBS57/TCRβ in CD45^+^IL-13GFP^+^ cells. (B) Quantitative PCR of DP, SP4, and thymic PBS57^+^ iNKT cells. Error bars indicate SEM. (C) WT thymocytes stimulated with PMA/Ionomycin. (Left) Gating on iNKT cells. (Right) IL-4/IL-13 production by iNKT cells. (D) Thymus sections of WT and *CD1d^−/−^* mice, and quantitation of conventional SP4 in grafts. Bars, 100 μm. (A–D) Data typical of at least three experiments, and six mice of each strain. (E) Thymic-resident iNKT cells identified in transplants as CD45.1^+^CD45.2^−^ cells. iNKT cell frequency in thymic-resident cells is shown with IL-4/IL-13 production after stimulation. Data represent four experiments; *n* = 18. Error bars indicate SEM. A Mann-Whitney nonparametric *U* test was performed. **, P < 0.01.

To examine the mechanism by which type-2 IL-4R signaling influences thymic egress, we used IL-13GFP reporter mice (Neill et al., 2010). We identified a small subset of CD45^+^IL-13GFP^+^ cells in the adult thymus at steady state (Fig. 5 A). Phenotypic analysis showed most of these cells were mCD1d-PBS57^+^ iNKT cells (Fig. 5 A; Liu et al., 2006; Rossjohn et al., 2012; White et al., 2014). Importantly, we also detected expression of both *Il4* and *Il13* mRNA in mCD1d-PBS57^+^ iNKT cells (Fig. 5 B) and a dominant IL-4^+^IL-13^+^ iNKT cell population after in vitro stimulation (Fig. 5 C). Interestingly, although earlier studies indicate that IL-4 production by thymic iNKT cells occurs in the steady state (Lee et al., 2015), culturing thymocytes with Brefeldin A, followed by flow cytometric analysis, did not reveal IL-13 protein expression in iNKT cells (not depicted). Thus, although our data indicate that thymic iNKT cells express *Il13* mRNA and so may be primed to produce IL-13, their steady-state in vivo production of IL-13 protein may require their controlled stimulation by additional unknown interactions in the medulla that are absent from thymocyte suspensions. To directly assess the requirement for iNKT cells in thymic egress, we analyzed *CD1d*^−/−^ mice (Mendiratta et al., 1997). Similar to *Il4ra^−/−^* mice, we saw mTEC-free areas containing SP thymocytes, accompanied by a significant increase in the most mature CD62L^+^HSA^−^ SP4 T-conv thymocytes (Fig. 5 D). Together with the findings of others on intrathymic IL-4 production (Lee et al., 2015), these findings suggest that iNKT cells represent a cellular source of IL-4Rα ligands and that these cells are required to promote thymic egress of αβT cells.

Intrathymic CD1d-restricted iNKT cells are heterogeneous and include long-term resident cells of unknown function that can be identified as persisting, donor-derived iNKT cells within lymphoid thymus transplants (Berzins et al., 2006). To examine the possible relevance of these cells to thymic egress, we transplanted lymphoid CD45.1^+^ thymus lobes into CD45.2^+^ mice and, after 6 wk, identified long-term, thymic-resident iNKT cells as CD45.1^+^ mCD1d-PBS57^+^ cells (Fig. 5 E). Interestingly, and in agreement with studies identifying the potent cytokine secretion ability of long-term, thymic-resident iNKT cells (Berzins et al., 2006), most CD45.1^+^ iNKT cells remaining within the thymus grafts produced IL-4 and IL-13 after stimulation (Fig. 5 E), suggesting that thymic-resident iNKT cells are an intrathymic source of cytokine ligands for the type-2 IL-4R.

By investigating the specialization of thymic stroma, we show that TECs express the type-2 IL-4R, which is functionally active in response to its ligands IL-4 and IL-13. That *Il4ra^−/−^* mice display multiple and specific medullary defects, including the intrathymic accumulation of mature CD4^+^ thymocytes and diminished numbers of RTE, points strongly toward a role for the type-2 IL-4R in controlling thymic exit. Importantly, although our findings show that IL-4Rα is expressed by TECs, additional non-TEC stroma may also express the type-2 IL-4R and contribute to regulation of thymic output. Whether its role described here is exclusive to TECs or not, the identification of IL-4Rα as a new regulator of thymus emigration reveals a novel mechanism by which thymic stroma influences thymocyte development and represents an important step in understanding late-stage thymus function. Relevant to that, although experiments performed here used BALB/c mice that are biased toward type-2 cytokine production, we also saw SP thymocyte accumulations in IL-13–deficient C57BL/6 mice (Fig. S2 C), suggesting that the influence of the type-2 IL-4R in thymic emigration extends beyond the BALB/c strain.

How the type-2 IL-4R controls thymic emigration of SP thymocytes is unclear. However, comparable levels of S1P_1_ and CD69 in WT and *IL-4ra^−/−^* SP4 thymocytes and successful blockade of emigration in *Il4ra^−/−^* mice by FTY720 argue that S1P-mediated emigration remains active. In addition, although blockade of S1P-mediated migration promotes T-lymphopenia (Zamora-Pineda et al., 2016), *Il4ra^−/−^* deficiency does not. Although the reasons for this difference are not clear, it perhaps indicates the influences of IL-4Rα and the S1P–S1P_1_ axis on thymic emigration are distinct. Interestingly, given that RTE in *Il4ra^−/−^* mice show increased maturity as indicated by diminished Rag2GFP levels compared with WT counterparts, it may be that type-2 IL-4R signaling controls the length of time mature thymocytes spend within the medulla to ensure a correct program of postselection maturation takes place, which may then determine the rate and efficacy of emigration. Interestingly, we found that IL-4/IL-13 stimulation of TEC induced expression of the chemokines CCL21 and CXCL10. Although CCL21 is a ligand for CCR7, a known regulator of thymic exit (Ueno et al., 2002), CXCL10 is a ligand for CXCR3, which controls intrathymic retention of iNKT cells (Drennan et al., 2009). These findings raise the possibility that type-2 IL-4R signaling in the thymus influences egress via multiple chemotactic mechanisms. First, it may control the production of chemokines that directly regulate the emigration of mature CCR7^+^ SP thymocytes. Second, as part of a positive feedback loop, it may boost TEC production of CXCL10, which acts to retain CXCR3^+^ iNKT cells in the thymus, where they can continue to trigger type-2 IL-4R signaling. Finally, thymocytes accumulating within the thymic PVS may also suggest that chemokines and/or other migratory factors controlled by IL-4Rα signaling are required for entry into the circulation.

That mTEC^lo^ express the type-2 IL-4R may also be significant in explaining its role in thymic egress. Indeed, mTEC^lo^ express CCL21 (Lkhagvasuren et al., 2013), which we show here can be induced by IL-4/IL-13, and its receptor (CCR7) is expressed by SP thymocytes and RTE (Ueno et al., 2002; Cowan et al., 2013, 2016). Interestingly, LTβR also regulates both thymocyte emigration and CCL21^+^mTEC^lo^ (Boehm et al., 2003; Lkhagvasuren et al., 2013) and, as with *Il4ra^−/−^* mice, defective thymus egress in *Ltbr*^−/−^ mice does not cause T lymphopenia (Boehm et al., 2003). However, notable differences exist between *Ltbr^−/−^* and *Il4ra^−/−^* mice. For example, although reduced mTEC may explain altered thymic egress in *Ltbr^−/−^* mice (Boehm et al., 2003; Lkhagvasuren et al., 2013), that cannot be the case for *Il4ra^−/−^* mice, in which TEC development is normal. Altered mTEC development and gross medulla disorganization in *Ltbr^−/−^* might also explain why intrathymic accumulations of thymocytes are more obvious in thymic sections of *Il4ra^−/−^* mice, where mTEC development and gross medullary architecture are normal. Finally, although expression of LTβR ligands maps to conventional SP thymocytes (Boehm et al., 2003; White et al., 2010), the cytokines that trigger type-2 IL-4R signaling for thymic egress are produced by iNKT cells, including long-term, thymic-resident cells. Thus, the type-2 IL-4R represents an important regulator of thymic microenvironments that enables innate T cells to regulate the thymic egress of conventional αβT cells.

## Materials and methods

### Mice

The following mouse strains on a BALB/c background were used at 8–12 wk of age: *Cd1d*^−/−^ (Mendiratta et al., 1997), *Il4ra^−/−^* (Mohrs et al., 1999), IL-13GFP (Neill et al., 2010). WT BALB/c littermates were used as controls. To examine thymocyte egress, we crossed *IL-4Ra^−/−^* mice to Rag2GFP mice (Yu et al., 1999). CD45.2^+^ C57BL/6, and congenic CD45.1^+^ BoyJ mice were used for thymus transplant experiments. For the generation of embryos, the day of vaginal plug detection was designated day 0. C57BL/6 IL-13^GFP/GFP^ mice were also used for analysis of thymus sections. Husbandry, housing, and experimental methods involving mice were performed at the Biomedical Services Unit at the University of Birmingham, in accordance with local ethical review panel and national Home Office regulations.

### Antibodies and cell sorting

Thymocyte and splenocyte suspensions were stained using antibodies to the following cell surface markers; CD4 Brilliant Violet (BV) 711 (RM4-5; BioLegend), CD8 BV785 (53-6.7; BioLegend), CD25 Alexa Fluor 700 (PC61; BioLegend), CD62L BV510 (MEL-14; BioLegend), TCR-β APC eFluor 780 (eBioscience), Foxp3 PE (fjk-16s; eBioscience), CD44 PE Cy-7 (IM7; eBioscience), CD69-APC (H1.2F3; eBioscience), HSA PerCp cy5.5 (M1/69; eBioscience), and PBS-57 421, the latter to detect iNKT cells (National Institutes of Health Tetramer Core Facility). Enzymatically digested thymic lobes were stained with antibodies to the following; CD80 BV605 (16-10A1; BioLegend), EpCAM-1 PerCp cy5.5 (G8.8; eBioscience), CD45 APC eFluor 780 (A20; eBioscience), and CD80 FITC (16-10A1; eBioscience). Biotinylated *Ulex europaeus* agglutinin-1 (Vector Laboratories) was detected using streptavidin PE Cy7 (eBioscience). All data were acquired using a BD LSR Fortessa with FACSDiva6.2 software, and analysis was performed using FlowJo software (Tree Star). Forward and side scatter gates were set to exclude nonviable and aggregated cells. TEC populations were sorted from digested preparations of adult mouse thymus (White et al., 2014), and the following phenotypes were sorted: mTEC^hi^ (CD45^−^EpCAM-1^+^UEA-1^+^MHCII^hi^CD80^+^), mTEC^lo^ (CD45^−^EpCAM-1^+^UEA-1^+^MHC II^lo^CD80^lo^), and cTEC (CD45^−^EpCAM-1^+^UEA-1^−^MHCII^+^). CD4^+^CD8^+^ and CD4^+^CD8^−^ thymocytes and thymic TCRβ^+^PBS57^+^ iNKT cells were sorted from mechanically prepared thymocyte suspensions and B cells (CD3^−^B220^+^) from splenocyte suspensions, all from 8–12-wk-old adult mice. All sorting (White et al., 2014) was performed on a MoFlo XDP (Beckman Coulter) or FACS Aria Fusion 1 (BD); in all cases, purity was checked postsorting and was typically >98%.

### Thymocyte stimulation and cytokine production

Thymocyte suspensions were cultured for 1 h in the presence or absence of 1.5 µM ionomycin and 50 µg/ml PMA (Sigma-Aldrich), then, for a further 2 h, with the addition of 10 μg/ml Brefeldin A (Sigma-Aldrich). Cells were prepared per manufacturer’s instructions for intracellular cytokine analysis using the BD cytofix/cytoperm kit and anti–IL-4 APC (11B11) and anti–IL-13 PE (eBio13A; eBioscience).

### FTOC

Embryonic thymus lobes at E15 (d 15) of gestation were placed in 1.35 mM 2dGuo, as previously described (Cowan et al., 2013). 2dGuo FTOC were then either transplanted in vivo or were used for in vitro experiments involving a further 4-d stimulation in the presence or absence of 100 µg/ml recombinant IL-4 (BioLegend) and IL-13 (PeproTech).

### Confocal microscopy

Both freshly isolated thymic tissue and thymus grafts were mounted in OCT and snap-frozen in liquid nitrogen before cryosectioning (Cowan et al., 2013). Antibodies used for immunolabeling of tissue sections were anti-CD4 Alexa Fluor 647 (RM4-5; BioLegend), anti-CD8 biotin (53–6.7, eBioscience), anti-CD31 FITC (PECAM-1; eBioscience), streptavidin Alexa Fluor 555 (Thermo Fisher Scientific), ERTR7 and ERTR5 (van Vliet et al., 1985), and goat anti-rat IgM Alexa Fluor 488 (Thermo Fisher Scientific). Analysis was performed using a ZEISS LSM 780 confocal microscope and ZEISS Zen Black software. 

### Thymus transplantation

Freshly isolated lymphoid E18 CD45.1^+^ thymus lobes were transplanted under the kidney capsule of congenic CD45.2^+^ mice and recovered after 6 wk to analyze iNKT cells. To examine the requirement for thymic stromal expression of IL-4Rα, BALB/c WT or *Il4ra^−/−^* 2dGuo-treated FTOC was transplanted into BALB/c nude mice for 10 wk. In both cases, grafting was performed as described (Cowan et al., 2013).

### Quantitative PCR

FACS-sorted cell populations were analyzed for mRNA expression of the indicated genes by qPCR exactly as described (Cowan et al., 2013). Primer sequences are as follows: β-actin QuantiTect Mm *Actb* 1SG Primer Assay (QT00095242; QIAGEN); *Foxo1* forward, 5′-TGTCAGGCTAAGAGTTAGTGAGCA-3′ and reverse, 5′-GGGTGAAGGGCATCTTTG-3′; *Klf2* forward, 5′-CTCAGCGAGCCTATCTTGCC-3′ and reverse, 5′-CACGTTGTTTAGGTCCTCATCC-3′; *S1pr1* forward, 5′-AAATGCCCCAACGGAGACT-3′ and reverse, 5′-CTGATTTGCTGCGGCTAAATTC-3′; *Ccr7* forward, 5′-CTAGCTGGAGAGAGACAAGA-3′ and reverse, 5′-TATCCGTCATGGTCTTGAGC-3′; *Il4ra* forward, 5′-ACACTACAGGCTGATGTTCTTCG-3′ and reverse, 5′-TGGACCGGCCTATTCATTTCC-3′; *Il13ra1* forward, 5′-ATGCTGGGAAAATTAGGCCATC-3′ and reverse, 5′-ATTCTGGCATTTGTCCTCTTCAA-3′; *Il13ra2* forward, 5′-TGGCAGTATTTGGTCTGCTCT-3′ and reverse, 5′-CAAGCCCTCATACCAGAAAAACA-3′; *Il2rg* forward, 5′-CTCAGGCAACCAACCTCAC-3′ and reverse, 5′-GCTGGACAACAAATGTCTGGTAG-3′; *Il4* forward, 5′-ATTTTGAACGAGGTCACAGGAGAAG-3′ and reverse, 5′-ACCTTGGAAGCCCTACAGACGAG-3′; *Il13* forward, 5′-TGAGCAACATCACACAAGACC-3′ and reverse, 5′-GGCCTTGCGGTTACAGAGG-3′; *Ccl21* forward, 5′-ATCCCGGCAATCCTGTTCTC-3′ and reverse, 5′-GGGGCTTTGTTTCCCTGGG-3′; *Cxcl10* forward, 5′-CCAAGTGCTGCCGTCATTTTC-3′ and reverse, 5′-GGCTCGCAGGGATGATTTCAA-3′.

In all cases, mRNA levels were normalized to β-actin; fold levels represent the means (±SEM) of replicate reactions, and data are typical of at least two independently sorted biological samples.

### FTY720 treatment

1 mg/kg FTY720 (Sigma-Aldrich) was administered i.p. daily for 3 d, with mice culled on the fourth day and thymocytes analyzed by flow cytometry.

### Labeling of SP thymocytes in the PVS

1 µg of PE-labeled anti-CD4 (clone GK1.5; eBioscience) was i.v. injected into mice (Zachariah and Cyster, 2010), which were sacrificed 3 min later. Thymocyte suspensions were costained with anti-CD8, and the number of CD4PE IV^+^ CD8^−^ cells was determined by flow cytometry.

### Quantitation of medullary epithelial-free areas

Tissue sections were randomly taken from throughout the thymus (3–4 per mouse), and the number of mTEC-free areas was assessed from images taken of the whole thymus section. The size and the contribution of the areas were calculated using ZEISS Zen Black software. To calculate areas within 100 μm of the CMJ, a line was drawn 100 μm from the CMJ.

### Phospho-STAT6 analysis in TEC

BALB/c 2dGuo lobes were disaggregated and depleted of residual CD45^+^ cells using Dyna Beads (Thermo Fisher Scientific). Cell suspensions were stimulated with 200 ng/ml of IL-4 (BioLegend) and IL-13 (PeproTech) for 30 min at 37°C. Cells were prepared per manufacturer’s instructions for intracellular antigens protocol C (eBioscience) and labeled using anti–phospho-STAT6 PE (CH12S4N; eBioscience).

### Statistical analysis

Prism (GraphPad Software) was used to perform all statistical analyses. The Mann-Whitney nonparametric *U* test was used, and graphs were annotated with the following markers to indicate significance: *, P < 0.05; **, P < 0.01; ***, P < 0.001; and ****, P < 0.0001. Nonsignificant differences were not specified. In all figures, bar charts and error bars represent the means ± SEM, respectively.

### Online supplemental material

Fig. S1 shows flow cytometric analysis of TEC populations in adult WT and *Il4ra^−/−^* mice. Fig. S2 shows analysis of thymus tissue from *Il13^−/−^*, *Il4^−/−^* (BALB/c background), and C57BL/6 *Il13*^−/−^ (C57BL/6) mice by flow cytometry (CD62L and HSA in SP4 thymocytes) and confocal microscopy (CD4, CD8, and ERTR5).

## Supplementary Material

Supplemental Materials (PDF)
