# Supplementary material for: A type 2 cytokine axis for thymus emigration
Source: J Exp Med. 2017 Aug 7;214(8):2205–16. doi: 10.1084/jem.20170271 (PMC5551576; doi:10.1084/jem.20170271)
Supplement: Supplemental Materials (PDF) [file JEM_20170271_sm.pdf]

The Journal of Experimental Medicine

SUPPLEMENTAL MATERIAL

White et al., https://doi.org/10.1084/jem.20170271

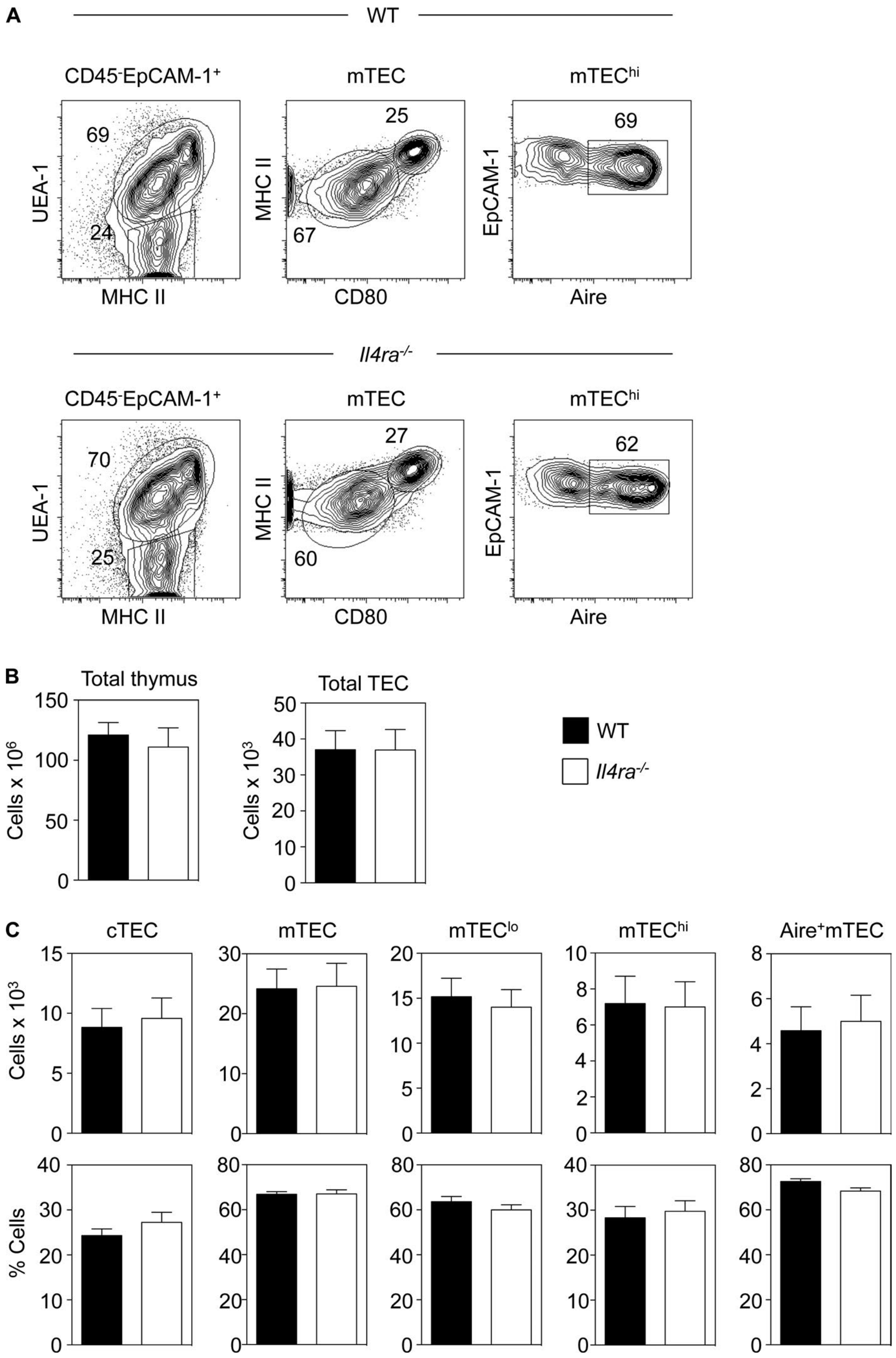

Figure S1. **TEC populations in *Il4ra*$^{-/-}$ mice.** (A) CD45-depleted thymic stromal preparations from WT (top) and *Il4ra*$^{-/-}$ (bottom) mice, after gating on EpCAM1$^+$ TEC. mTEC are identified as EpCAM1$^+$MHCII$^+$UEA1$^+$ cells and mTEC^{hi} as EpCAM1$^+$UEA1$^+$MHCII^{hi}CD80^{hi} cells. (B) Total thymus cellularity after digestion and the total TECs. (C) Quantitative analysis of TEC populations identified in A. Data are typical of at least three separate experiments, where *n* = 9 for both WT and *Il4ra*$^{-/-}$ mice.

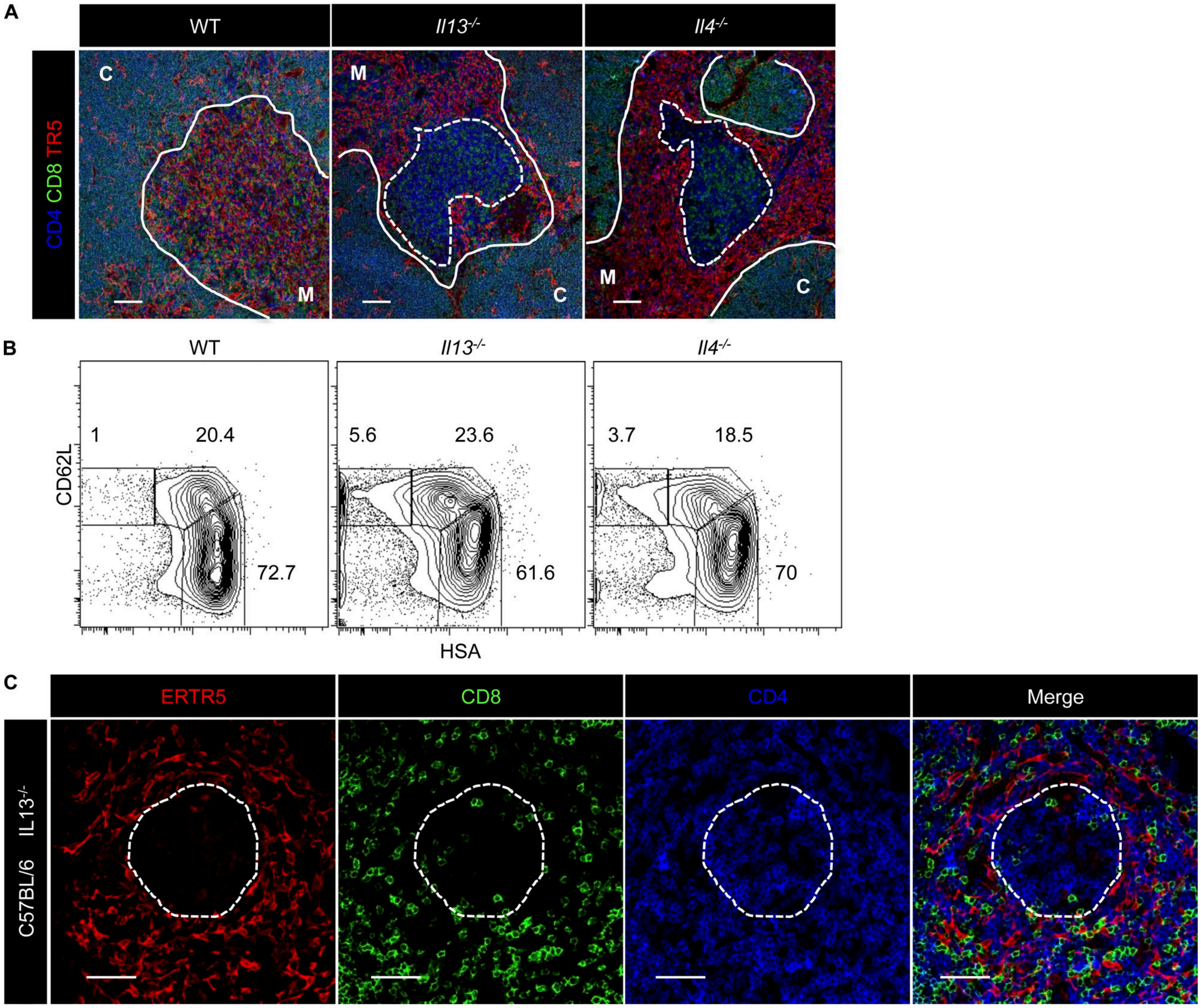

Figure S2. **Thymocyte accumulations in cytokine-deficient mice.** (A) Confocal images of frozen thymus sections of WT, *Il13*$^{-/-}$, and *Il4*$^{-/-}$ mice on a BALB/c background. TR5 identifies mTEC (red), CD4 is in blue, and CD8 in green. Solid line denotes CMJ, dotted line denotes boundaries of SP thymocyte accumulations. Bars, 100 µm. (B) CD62L/CD69 expression on SP4TCRβ^{+} thymocytes from the indicated mouse strains. (C) Confocal images of a frozen thymus section of tissue from *Il13*$^{-/-}$ mice on a C57BL/6 background. ERTR5 is used to identify mTEC (red), CD4 is in blue, and CD8 in green. Dotted lines denote boundary of SP thymocyte accumulations. Bars, 50 µm. Data are typical of at least two separate experiments, where *n* = 3 for WT and knockout strains.
